# Municipal spending on school health services is unequally associated with dual-factor mental health profiles: a latent profile study of 63,000 Swedish adolescents, 2006–2024

**DOI:** 10.1186/s12889-026-30221-2

**Published:** 2026-11-06

**Authors:** Rebecka Persson, Karl Wennberg

**Affiliations:** Center for Educational Leadership & Excellence, Stockholm School of Economics, PO Box 6501, Stockholm, 11383 Sweden

**Keywords:** Adolescent mental health, Dual-factor model, School health services, Precision public health, Well-being, Sweden

## Abstract

**Background:**

Adolescent mental health has declined internationally, prompting increased investment in school-based services. However, whether such investments benefit all students equally remains unclear. We examined whether municipal spending on school health services is associated with adolescent mental health in Sweden, and whether this association differs across distinct profiles. These profiles, defined using the dual-factor model, separate adolescents’ psychiatric symptoms from their school well-being.

**Methods:**

Data were drawn from Stockholmsenkäten (the Stockholm Survey), a biennial population-based survey of 9th-grade students (approximately 15 years) in Region Stockholm, Sweden. The analytic sample comprised 63 190 students across 26 municipalities surveyed between 2006 and 2024. Latent profile analysis identified four mental health profiles based on psychiatric symptoms and school well-being: Complete Mental Health, Troubled (high symptoms, low well-being), Vulnerable (low symptoms, low well-being), and Symptomatic but Content (high symptoms, adequate well-being). Municipal spending on school health services (per student, lagged two years) was decomposed into within-municipality (year-to-year change) and between-municipality (stable differences) components, both entered as predictors in the model. A multilevel multinomial logistic regression (students nested within municipalities) estimated associations between spending and profile membership, controlling for student gender and survey year.

**Results:**

Complete Mental Health declined from 55.6% (2006) to 45.4% (2024), while the Troubled profile more than doubled (3.4% to 7.2%). The Vulnerable profile remained stable at approximately 17%. Within-municipality spending increases were associated with 16% lower odds of the Troubled profile (OR = 0.84, 95% CI 0.73–0.95, *p* = .007) but only a borderline, non-significant association with the Vulnerable profile (OR = 1.08, 95% CI 0.99–1.17, *p* = .084). Spending was associated with 9% higher odds of the Symptomatic but Content profile (OR = 1.09, 95% CI 1.01–1.17, *p* = .026), possibly reflecting a compositional shift from the Troubled profile, as students who retain psychiatric symptoms nonetheless regain adequate school well-being. Between-municipality differences in average spending were largely unrelated to profile membership.

**Conclusions:**

Increased municipal spending on school health services was associated with a lower prevalence of the symptomatic Troubled profile, consistent with services reaching students whose distress is visible, but showed no comparable association for the Vulnerable profile, that is, students whose difficulties manifest as low school well-being without psychiatric symptoms. From a precision public health perspective, these findings suggest that school-based services may need to complement their symptom-oriented focus with systematic attention to school well-being in order to reach the Vulnerable subgroup, who comprise roughly one in six adolescents and showed no measurable association with spending in this study.

## Background

The mental health of children and adolescents has emerged as a critical public health concern internationally. Recent epidemiological data reveal a marked decline in mental health among youth, with increasing rates of psychiatric service utilization and self-reported psychological distress [1–4]. These trends carry substantial implications for educational systems, with mental health problems associated with reduced academic achievement, school absence, and impaired social functioning [5, 6]. With the decline in youth mental health, public expenditure on school-based health services has increased in many high-income countries, including Sweden [7, 8]. In line with research on precision in public health and psychological theory about mental health, we apply a heterogeneity perspective to the effectiveness of school health service expenditures. Children’s mental health must be understood both as the absence of psychiatric symptoms and as the presence of enough well-being to support learning and social participation, because these are distinct dimensions that each uniquely predict developmental outcomes [9, 10], a dual conceptualization formalized in the dual-factor model of mental health, which we describe in detail below. Using both perspectives allows schools to identify problems earlier and support students who struggle functionally even before clinical symptoms appear. A dual conceptualization of children’s mental health implies that school mental health systems must address both diagnosable symptoms and impairments in well-being, since each dimension independently predicts academic, social, and long-term psychiatric outcomes. Effective investments must support not only clinical services but also preventive initiatives that enhance well-being and functioning at the student population level.

With data from 63 190 students from 2006 to 2024 in the Stockholm region, we test whether higher municipal spending on school health services is associated with mental health in youth. We do not test for this in terms of general psychiatry or well-being, but in terms of four mental health profiles, stemming from the dual factor model of mental health. Although our analyses are situated in a Swedish municipal context, the heterogeneity perspective we apply, distinguishing students whose distress is symptomatic from those whose struggles manifest as diminished well-being, is portable to any educational system in which school-based mental health services operate as a primary point of contact for adolescents, including most high-income and many middle-income countries.

### School health expenditure effectiveness

A growing body of evidence suggests that school-based and community interventions can improve adolescent mental health outcomes [5, 11, 12]. In a systematic review of cost-effective mental health interventions by Le et al. [13], the school-based screening and psychological interventions were the most cost-effective strategy for preventing mental disorders amongst children and adolescents. Meanwhile, parenting- and workplace interventions presented good evidence to be effective for promotion of mental health and well-being. Importantly, targeted interventions rather than general ones were likely to be more cost-effective. This suggests that we also need targeted evaluations to understand if mental health expenditure has subgroup recipients or if it prevents mental health problems in all students. However, the distinction between targeted and universal approaches may also adhere to an understanding of schools as a social context of children and youth that is significant to students with mental health problems. A meta-analysis of whole-school interventions found that programs addressing the social and emotional development of all students, rather than selectively targeting those with identified problems, produced meaningful improvements in well-being and reduced emotional difficulties [14]. This aligns with conceptual work positioning school well-being as a distinct construct, shaped by the school environment’s capacity to meet students’ needs for safety, belonging, and meaningful participation [15, 16]. If school health services operate primarily through symptom detection and clinical referral, they may effectively reach students whose distress is visible while failing to support those whose diminished well-being has not yet become symptomatic. Given that intervention effectiveness may depend on the match between service model and student profile, the present study takes a heterogeneity perspective, which considers how spending may differentially affect students across distinct mental health profiles.

Precision public health seeks to deliver the right intervention to the right subgroup at the right time, moving beyond population-average effects to ask which groups benefit from an intervention and which do not [17, 18]. Subgroup analysis is therefore fundamental to it [5]. In this study, subgroups emerge from a theoretical model of psychological functioning. The dual-factor model classifies individuals into four subgroups derived from the cross-classification of psychological distress and subjective well-being [10, 19, 20].

### The dual factor model of mental health

Traditional approaches to youth mental health have predominantly focused on the presence or absence of psychopathology. However, this unidimensional perspective fails to capture the full complexity of mental health. The dual-factor model of mental health, proposed by Greenspoon and Saklofske [19] and further developed by Keyes [9] and Suldo and Shaffer [10], conceptualizes mental health as comprising two related but distinct dimensions: psychopathology (the presence of psychiatric symptoms) and subjective well-being (the presence of positive psychological functioning). This framework aligns with the definition adopted by WHO’s (2021) definition of mental health as “a state of mental well-being that enables people to cope with the stresses of life, realize their abilities, learn and work well, and contribute to their community” [21]. National authorities recognizes both well-being and mental illness as constituent elements of mental health.

The dual-factor model identifies four distinct mental health profiles based on individuals’ standings on both dimensions [20, 22]. Children and adolescents classified as having *Complete Mental Health* exhibit high well-being and low psychopathology. The *Troubled* group presents with both low well-being and elevated psychiatric symptoms. Critically, the model reveals two additional profiles that remain invisible in traditional symptom-focused assessments: *Vulnerable* youth who report low well-being despite the absence of psychiatric symptoms, and *Symptomatic but Content* individuals who maintain adequate well-being despite experiencing psychiatric symptoms. Research demonstrates that vulnerable youth exhibit academic outcomes comparable to those with clinical symptomatology [10, 23], underscoring the importance of assessing both dimensions.

A key question in dual-factor research concerns the stability and variability of mental health profiles over time. A recent cross-sectional community study in the Gotland region of Sweden found a very high (47.5%) rate of Vulnerable and Troubled (13.9%) students among 1 833 surveyed students in grades 7–12. However, other studies indicate that many adolescents do not maintain stable profile classifications over time, suggesting substantial potential for change [24]. This instability may reflect both developmental processes and environmental influences, including school-related factors. Indeed, recent research indicates that schools account for a small but significant proportion of variance in student mental health outcomes [25, 26], and that school climate represents a modifiable protective factor [27]. These findings suggest that educational environments may differentially shape the manifestation of dual-factor profiles among students.

In Sweden, school health services represent a key point of contact for monitoring and supporting student mental health [28]. School health teams, comprising school nurses, physicians, psychologists, counselors, and special educators, are mandated to work preventively and health-promotively. Effectiveness of such health-promoting efforts depends not only on resources, but also on how school leadership organizes and prioritizes this work [29]. However, resources allocated to school health services vary considerably across municipalities. In the Stockholm region, per-student expenditure on school health services differs substantially between municipalities and over time, creating natural variation in the availability of mental health support. Whether such resource differences are associated with the distribution and stability of dual-factor mental health profiles remains unexplored.

Studies using the dual-factor model have shown that the Vulnerable and Symptomatic but Content profiles are common, that they carry academic and psychosocial risks comparable to or exceeding symptom-only classifications, and that profile membership is only moderately stable over adolescence [10, 20, 22–24]. Despite this growing evidence, we know of no studies that have examined whether resource allocation, specifically investment in school health services, is associated with the prevalence, distribution, or temporal stability of mental health profiles among students. This gap is significant from a public health perspective, as school health services represent a modifiable and potentially scalable intervention target [30]. Understanding whether differential resource allocation is associated with mental health profile variability could inform evidence-based decisions about school health investments.

### The present study

The present study addresses this gap by examining associations between per-student expenditure on school health services and the distribution of dual-factor mental health profiles among students in Stockholm municipalities. Using lagged spending data (spending at year *t* − 2 predicting profiles at year *t*), we distinguish within-municipality effects (changes in spending over time) from between-municipality effects (stable differences in average spending), providing a stronger basis for inference about within-municipality change, while remaining an observational design.

Specifically, we: [1] characterize the prevalence and temporal trends of dual-factor mental health profiles in a population-based sample of 9th-grade students from 2006 to 2024; [2] examine whether within-municipality changes in spending on school health services are associated with the distribution of mental health profiles, controlling for time trends and stable between-municipality differences; and [3] assess whether the association between spending and profile membership differs across the four profiles; specifically, whether the association differs for the Troubled profile (characterized by visible psychiatric symptoms) versus the Vulnerable profile (characterized by low well-being without symptoms).

## Methods

### Participants and design

This study used data from The Stockholm survey, a biennial population-based survey administered to students in the greater Stockholm region in Sweden. The survey is coordinated by City of Stockholm and has been conducted since 2002, with consistent mental health items available from 2006 onwards. The analytic sample comprised 63 190 students in Grade 9 (approximately 15 years of age) across 26 municipalities, surveyed between 2006 and 2024 (10 waves). The sample was approximately balanced by gender, with 49.3% boys, 48.5% girls, and 0.8% identifying as non-binary (the latter category available from 2018 onwards). Stockholm municipality constituted approximately 90% of the sample (*n* = 56 813), with remaining municipalities contributing between 10 and 1 103 students each across the study period. Because one very large municipality (Stockholm) predominates alongside many much smaller ones, cross-sectional comparisons between municipalities are difficult to interpret; our analysis therefore emphasizes within-municipality change over time, with each municipality serving as its own comparison, rather than differences between municipalities (see Statistical Analysis).

The survey is administered in the classroom during regular lesson hours in the spring term, is answered anonymously and voluntarily, and has historically used a paper questionnaire returned in a sealed envelope, with electronic administration introduced in more recent waves. Because the survey is offered to all schools in participating municipalities rather than to a drawn sample, it approximates a census of enrolled Grade 9 students in participating municipalities in each wave; response rates have varied across waves (for example, in the City of Stockholm, approximately 78% in 2016 and 73% in 2018 [31]), with non-response arising primarily from student absence and from schools or municipalities that did not participate in a given wave. Because participation requires attendance on the survey day, students who are frequently absent, potentially including some with the highest levels of distress, may be underrepresented, an issue we revisit in the Limitations.

### Measures

#### Mental health indicators

Mental health was operationalized using the dual-factor model [10, 19], which conceptualizes mental health as comprising two related but distinct dimensions: psychopathology (psychiatric symptoms) and well-being, which in the present study was operationalized as school well-being (see below).

*Psychiatric symptoms* were measured using four items assessing frequency of: feeling sad without knowing why, feeling anxious or afraid without knowing why, feeling worthless, and feeling sluggish or listless. Items were rated on a 5-point scale (1 = *never* to 5 = *always*). A fifth item assessing sleep problems was initially considered but excluded from the final analysis due to poor differentiation across latent profiles and a temporal trend (improvement over time) inconsistent with other symptom indicators.

*School well-being* was measured using three items from a school satisfaction scale: thriving at school, finding schoolwork meaningful, and looking forward to lessons. Items were rated on a 4-point scale (1 = *strongly disagree* to 4 = *strongly agree*). One item (finding schoolwork meaningful) was reverse-coded. Items were recoded so that higher scores consistently indicated more frequent symptoms and higher well-being, respectively. All seven items were used as continuous indicators in the latent profile analysis. These items are drawn from the school domain of the Stockholm Survey instrument, which has been used extensively in Swedish adolescent research, and they correspond conceptually to the school-satisfaction and school-well-being dimensions described in established school-well-being frameworks [15].

It is important to note that the well-being dimension in the present study reflects school well-being (school satisfaction) rather than the global subjective well-being originally conceptualized in the dual-factor model. School well-being has been described as a distinct, school-shaped domain of adolescent well-being [15], and the school environment it captures is itself associated with students’ emotional health [27]. While narrower than global subjective well-being, it is conceptually aligned with the well-being pole of the dual-factor model. We regard school well-being as a theoretically appropriate and policy-relevant indicator in this context because it is the dimension most proximal to, and most plausibly modifiable by, school-based services. It is, nonetheless, a narrower construct than global well-being, and we therefore use the terms “school well-being” and “school satisfaction” throughout to keep this scope explicit; the Vulnerable profile identified here should accordingly be read as low school well-being in the absence of psychiatric symptoms. We return to the implications of this narrower operationalization in the Limitations.

#### Covariates

Gender was included as a covariate (female = 1, male = 0; non-binary students were excluded from the regression analysis due to small cell sizes, *n* = 532). Survey year was included as a continuous variable to control for secular trends in adolescent mental health.

#### School health services spending

Municipal spending on school health services (elevhälsa) per student was obtained from the Swedish Agency of Education’s annual public accounts. This standardized measure captures actual expenditures reported in municipal financial statements and includes costs for school nurses, school physicians, school psychologists, school counselors, and special educators. The measure covers the school health service as a whole. The accounts report a single figure for school health services and do not separate spending on students’ mental health from spending on their physical health or learning support; the measure is therefore not restricted to mental health services. Spending data was available for all years from 2002 to 2024. The way this expenditure is generated differs across municipalities and over time. In several municipalities, including the City of Stockholm, resources are distributed to schools through a needs-based allocation model in which the socioeconomic characteristics of the student body are an explicit parameter, so that schools serving more disadvantaged populations receive larger per-student allocations. Moreover, school health services are not always a separately ring-fenced budget line: in some municipalities the costs are held centrally, whereas in others they are embedded in the per-student allocation and the share devoted to school health is decided at the school level by the principal. This institutional heterogeneity means that the measure captures the resources devoted to school health services, but not the precise mechanism or level at which they are allocated, a point we consider when interpreting between-municipality differences and in the Limitations.

To establish temporal precedence and reduce the risk of reverse causality (i.e., municipalities increasing spending in response to observed mental health problems), spending was lagged by two years relative to the survey year. This two-year lag aligns with the biennial survey administration and allows time for resource investments to plausibly influence student outcomes.

### Statistical analysis

#### Latent profile analysis

Latent Profile Analysis (LPA) [32] was conducted in Mplus version 9 [33] to identify distinct subgroups of students based on their patterns of psychiatric symptoms and school well-being. A four-class solution was specified a priori based on the dual-factor model’s theoretical framework, which posits four profiles: Complete Mental Health (low symptoms, high well-being), Troubled (high symptoms, low well-being), Vulnerable (low symptoms, low well-being), and Symptomatic but Content (high symptoms, high well-being) [10].

Model estimation used maximum likelihood with robust standard errors (MLR), which is robust to non-normality. Due to the large sample size (*N* = 63 190) and potential for local maxima, initial analyses were conducted on a 20% random subsample (*n* = 12 642) to identify optimal starting values. The best-fitting solution from the subsample was then applied to the full sample to ensure stable convergence.

Model fit was evaluated using entropy, with values above 0.80 indicating good classification accuracy [34]. The final four-class solution demonstrated good classification quality (entropy = 0.853). Students were assigned to their most likely latent class based on posterior probabilities.

#### Multilevel regression analysis

To examine associations between municipal spending and mental health profile membership, we estimated a multilevel multinomial logistic regression model using generalized structural equation modeling (gsem) in Stata version 18 [35]. The model accounted for the hierarchical structure of the data, with students (Level 1) nested within municipalities (Level 2). A random intercept was specified for municipality to account for unobserved heterogeneity across municipalities.

To disentangle within- and between-municipality effects of spending [36], we decomposed the lagged spending variable into two components: [1] between-municipality spending, defined as the mean spending of each municipality across all study years (time-invariant), representing stable differences between municipalities in resource allocation; and [2] within-municipality spending, defined as the deviation of each year’s spending from that municipality mean (time-varying), representing year-to-year fluctuations in spending within municipalities.

This decomposition addresses a key limitation of observational studies: municipalities that spend more may differ systematically from those that spend less in ways that also affect student mental health. The within-municipality effect controls for these stable confounders by asking whether changes in spending within a given municipality over time are associated with changes in mental health profiles. This provides a stronger basis for inference than the between-municipality effect, although it does not by itself establish causality [37].

Spending was scaled to 1 000 Swedish SEK (approx. €100) units for interpretable coefficients. Complete Mental Health was specified as the reference category; thus, coefficients represent log-odds of belonging to each at-risk profile (Vulnerable, Troubled, or Symptomatic but Content) relative to Complete Mental Health.

### Missing data

Missing data on individual survey items were handled using full information maximum likelihood (FIML) in the LPA, which uses all available data and provides unbiased estimates under the assumption that data are missing at random [38]. For the multilevel regression analysis, cases with missing data on gender were excluded (*n* = 879, 1.4%), resulting in a final analytic sample of 62 311 students. The merge between survey data and municipal spending data resulted in complete matching for all students.

### Declaration of generative AI and AI-assisted technologies in the manuscript preparation process

During the preparation of this work the authors used Claude Sonnet 4.6 in order to assist with language editing and refinement of manuscript text. After using this tool/service, the authors reviewed and edited the content as needed and take full responsibility for the content of the published article.

## Results

### Latent profile analysis

A four-class solution demonstrated good classification quality (entropy = 0.853), exceeding the recommended threshold of 0.80 [34]. The four profiles aligned with the theoretical expectations of the dual-factor model and were labeled accordingly [10]. Table 1 presents the indicator means and class sizes for each profile.

**Table 1:** Latent profile indicator means and class sizes (*N* = 63 190)

Indicator	Complete MH	Symptomatic	Vulnerable	Troubled
	(*n = 32 264)*	(*n = 16 388)*	(*n = 10 825)*	(*n = 3 713)*
Psychiatric symptoms (1–5)				
Feeling sad	1.78	3.50	3.38	4.11
Feeling afraid/anxious	1.05	2.46	1.01	4.40
Feeling worthless	1.56	2.91	2.95	3.58
Feeling sluggish	1.83	2.94	3.12	3.50
School well-being (1–4)				
Thriving at school	3.53	3.08	2.96	2.78
Schoolwork meaningful	3.12	2.67	2.43	2.41
Looking forward to lessons	2.52	2.11	1.79	1.98

Higher scores on psychiatric symptoms indicate more frequent symptoms. Higher scores on well-being indicate better well-being. Entropy = 0.853

Complete *MH* Complete Mental Health, Symptomatic = Symptomatic but Content

*Complete Mental Health* (51.1%, *n* = 32 264) was the largest profile, characterized by low psychiatric symptoms (all means < 2.0 on the 5-point scale) and high school well-being (all means > 2.5 on the 4-point scale). *Symptomatic but Content* (25.9%, *n* = 16 388) showed moderate-to-elevated psychiatric symptoms alongside moderate school well-being. *Vulnerable* (17.1%, *n* = 10 825) exhibited moderate symptoms of sadness (M = 3.38), worthlessness (M = 2.95), and sluggishness (M = 3.12), but notably low anxiety (M = 1.01); this group reported the lowest school well-being across all indicators. *Troubled* (5.9%, *n* = 3 713), the smallest profile, showed uniformly high psychiatric symptoms, including the highest anxiety levels (M = 4.40).

An important distinguishing feature was anxiety: Vulnerable students reported anxiety levels nearly identical to the Complete Mental Health group (M = 1.01 vs. 1.05), while Troubled students reported dramatically elevated anxiety (M = 4.40). This pattern suggests that anxiety may differentiate students who are struggling but under the radar (Vulnerable) from those with more acute, visible distress (Troubled).

### Temporal trends in mental health profiles

Profile distributions changed substantially over the 18-year study period (see Table 2). The proportion of students classified as Complete Mental Health declined from 55.6% in 2006 to 45.4% in 2024, a decrease of 10.2% points. Conversely, the Troubled profile more than doubled in prevalence, from 3.4% in 2006 to 7.2% in 2024. The Symptomatic but Content profile also increased, from 23.1% to 29.5%.

**Table 2:** Mental health profile distribution by year (%)

Year	*n*	Complete MH	Symptomatic	Vulnerable	Troubled
2006	5 739	55.6	23.1	18.0	3.4
2008	6 425	55.8	23.5	16.2	4.5
2010	6 465	58.4	22.7	14.9	4.0
2012	6 150	56.4	23.1	17.0	3.5
2014	5 120	52.1	25.5	17.0	5.5
2016	6 350	51.8	25.7	15.8	6.7
2018	5 416	50.4	24.8	17.7	7.1
2020	6 368	46.2	28.4	17.9	7.5
2022	7 341	41.9	30.9	18.6	8.6
2024	7 816	45.4	29.5	18.0	7.2
Change		−10.2	+ 6.4	0.0	+ 3.8

Complete *MH*  Complete Mental Health, Symptomatic = Symptomatic but Content. Change = 2024 minus 2006

Notably, the Vulnerable profile remained remarkably stable throughout the study period, fluctuating narrowly between 14.9% and 18.6% across all waves of measurement. This stability indicates that the proportion of students experiencing low well-being without clinical symptoms has remained constant even as overall mental health has deteriorated.

The trajectory of decline accelerated around 2014–2016, with the steepest decreases in Complete Mental Health occurring between 2018 and 2022. [1] The 2022 wave showed the lowest proportion of Complete Mental Health (41.9%) and highest proportion of Troubled (8.6%), with a partial recovery observed in 2024.

### Gender differences

Substantial gender differences were observed in profile distributions (see Table 3). In 2024, 59.3% of boys were classified as Complete Mental Health compared to only 31.4% of girls, a difference of 27.9% points. Girls were overrepresented in all at-risk categories: approximately 2.8 times more likely to be classified as Troubled (10.3% vs. 3.7%), 1.2 times more likely to be Vulnerable (20.0% vs. 16.2%), and 1.9 times more likely to be Symptomatic but Content (38.4% vs. 20.7%).

**Table 3:** Mental health profile distribution by gender in 2024 (%)

Gender	*n*	Complete MH	Symptomatic	Vulnerable	Troubled
Boys	3 930	59.3	20.7	16.2	3.7
Girls	3 723	31.4	38.4	20.0	10.3
Non-binary	152	29.6	36.2	13.8	20.4
Gap (Girls − Boys)		−27.9	+ 17.7	+ 3.8	+ 6.6

Complete *MH*  Complete Mental Health, Symptomatic = Symptomatic but Content. Non-binary response option available from 2018 onwards

The gender gap widened over the study period. In 2006, the difference in Complete Mental Health between boys (68.8%) and girls (42.8%) was 26.0% points; by 2024, this gap had grown to 27.9% points. The increase in the Troubled category was more pronounced among girls (from 4.7% to 10.3%) than among boys (from 2.1% to 3.7%).

Students identifying as non-binary (assessed from 2018 onwards) showed the highest rates of distress. In 2024, only 29.6% of non-binary students were classified as Complete Mental Health, and 20.4% were classified as Troubled, nearly six times the rate observed among boys.

### Municipal variation

Profile distributions varied across municipalities. Among municipalities with adequate sample sizes (*n* > 200), the proportion of students classified as Complete Mental Health ranged from 42.3% to 54.1%. Mean spending on school health services ranged from 2 160 to 3 683 SEK per student across Stockholm region municipalities. Within Stockholm municipality, which comprised 90% of the sample, spending increased from 2 390 to 4 490 SEK between 2006 and 2024, an increase of 88%. Descriptively, this cross-sectional range was modest relative to the within-municipality change over time, and Complete Mental Health did not vary systematically with municipality size in a consistent direction; the largest and most urban unit, Stockholm municipality, fell within the same range as smaller suburban municipalities. Because Stockholm municipality students predominate in the overall sample, these between-municipality contrasts should be read cautiously, and our modelling therefore emphasizes within-municipality change rather than differences in municipal rank.

### Association between school health services spending and profiles

#### Descriptive associations

Raw associations between spending and profiles showed a counterintuitive pattern: students in the Troubled profile were in municipality-years with higher mean spending (M = 2 879 SEK) compared to Complete Mental Health students (M = 2 675 SEK). However, spending was highly correlated with survey year (*r* = .89), reflecting a secular increase in both spending and mental health problems over time. This confounding necessitated the decomposition of spending into within- and between-municipality components.

#### Multilevel multinomial regression

Results from the multilevel multinomial logistic regression are presented in Table 4. Complete Mental Health served as the reference category; coefficients represent odds ratios for membership in each at-risk profile relative to Complete Mental Health.

**Table 4:** Multilevel Multinomial Logistic Regression: Predictors of Mental Health Profile Membership

Predictor	Symptomatic but Content		Vulnerable		Troubled	
	OR	95% CI	OR	95% CI	OR	95% CI
Within-municipality spending	1.09*	[1.01, 1.17]	1.08	[0.99, 1.17]	0.84**	[0.73, 0.95]
Between-municipality spending	0.84*	[0.72, 0.99]	1.06	[0.84, 1.33]	1.10	[0.82, 1.49]
Girls (ref. boys)	3.52***	[3.38, 3.66]	2.22***	[2.12, 2.32]	4.01***	[3.72, 4.32]
Survey year (per year)	1.03***	[1.02, 1.04]	1.02***	[1.01, 1.03]	1.09***	[1.08, 1.11]

Reference category = Complete Mental Health. Odds ratios are per 1,000 SEK (≈ €100); within-municipality spending = deviation from the municipality mean, between-municipality spending = municipality mean across years, entered simultaneously. Models were additionally adjusted for self-reported sex (reference = boys) and survey year (linear term, odds ratio per year). *N* = 62,311 students in 26 municipalities

*OR*  odds ratio, *CI*  confidence interval

*p < .05. **p < .01. ***p < .001

*Within-municipality spending effects.* The within-municipality spending effect, representing the association between year-to-year changes in spending within a municipality and student profiles, showed a statistically significant protective association for the Troubled profile only. A 1 000 SEK (roughly €100) increase in spending above a municipality’s own average was associated with 16% lower odds of being classified as Troubled versus Complete Mental Health (OR = 0.84, 95% CI [0.73, 0.95], *p* = .007).

The association with the Vulnerable profile was of borderline significance and did not reach the conventional threshold (OR = 1.08, 95% CI [0.99, 1.17], *p* = .084); with a lower confidence limit at 0.99, this estimate is compatible with a small positive association and should not be read as evidence of no effect. Unexpectedly, the Symptomatic but Content profile showed a small positive association with within-municipality spending (OR = 1.09, 95% CI [1.01, 1.17], *p* = .026), possibly reflecting a partial compositional shift from the Troubled profile as students’ school well-being improved with greater service access.

#### Between-municipality spending effects

Between-municipality differences in mean spending were largely non-significant, except for the Symptomatic but Content profile, where municipalities with higher average spending had fewer Symptomatic students (OR = 0.84, 95% CI [0.72, 0.99], *p* = .033). This between-municipality estimate should be interpreted with particular caution, because stable differences between municipalities may reflect unmeasured characteristics that also affect adolescent mental health, for example, the socioeconomic composition of the student population, the proportion of residents born abroad, urbanicity, and differences in how municipalities organize and account for school health services. We deliberately did not adjust for municipal socioeconomic status: because per-student resources are, in several municipalities, allocated to schools through a needs-based formula in which socioeconomic disadvantage is an explicit parameter, municipal socioeconomic status is a determinant of the exposure itself, so that conditioning on it would risk over-control rather than removing confounding variability (see Limitations).

#### Covariates

Consistent with the descriptive results, female sex and later survey year were associated with higher odds of all at-risk profiles relative to Complete Mental Health (Table 4). Girls had higher odds than boys of being classified as Vulnerable (OR = 2.22, 95% CI [2.12, 2.32]), Symptomatic but Content (OR = 3.52, 95% CI [3.38, 3.66]), and Troubled (OR = 4.01, 95% CI [3.72, 4.32]). Each additional survey year was associated with approximately 2% to 9% higher odds of the at-risk profiles (Vulnerable OR = 1.02, 95% CI [1.01, 1.03]; Symptomatic but Content OR = 1.03, 95% CI [1.02, 1.04]; Troubled OR = 1.09, 95% CI [1.08, 1.11]; all *p* < .001).

### Robustness analyses

Sensitivity analyses examined whether results were robust to sample composition. As seen in Table 5, the protective association with the Troubled profile was replicated when excluding small municipalities (OR = 0.82, 95% CI [0.72, 0.93], *p* = .003). In Stockholm municipality alone, which comprised 90% of the sample, the effect was stronger (OR = 0.72, 95% CI [0.61, 0.86], *p* < .001). However, when Stockholm was excluded, the effect was no longer significant (OR = 0.97, 95% CI [0.79, 1.21], *p* = .813), though statistical power was limited (*n* = 6 265). Across all specifications, the Vulnerable profile showed no significant association with spending

**Table 5:** Robustness analyses: within-municipality spending effects on mental health profiles

Model	*N*	Symptomatic		Vulnerable		Troubled	
		OR [95% CI]	*p*	OR [95% CI]	*p*	OR [95% CI]	*p*
Main model	62 311	1.09 [1.01, 1.17]	0.026	1.08 [0.99, 1.17]	0.084	0.84 [0.73, 0.95]	0.007
Stockholm only	56 046	1.03 [0.93, 1.13]	0.597	1.07 [0.96, 1.19]	0.200	0.72 [0.61, 0.86]	< 0.001
Excluding Stockholm	6 265	1.14 [1.00, 1.29]	0.045	1.06 [0.92, 1.22]	0.446	0.97 [0.79, 1.21]	0.813
Excluding small municipalities	61 838	1.09 [1.01, 1.18]	0.023	1.07 [0.99, 1.17]	0.104	0.82 [0.72, 0.93]	0.003

Reference category = Complete Mental Health. Within-municipality spending = effect of a 1,000 SEK increase above a municipality’s own mean, controlling for between-municipality differences, sex, and survey year. Stockholm municipality only: single-level multinomial logistic regression (random intercept not estimable with one municipality). Excluding Stockholm municipality: multilevel model with 25 municipalities. Excluding small municipalities: excludes 11 municipalities with *n* < 100.

*OR*  odds ratio, *CI*  confidence interval

The sensitivity of results to lag length was also examined. Models were re-estimated using spending lagged one, three, and four years prior to the survey, in addition to the primary two-year lag. The protective association between municipal spending and Troubled profile membership was statistically significant across all four lag specifications, with odds ratios ranging from 0.737 to 0.888, corresponding to reductions in odds of 11% to 26%. The effect was most pronounced at the two-to-three-year lag window, with the three-year lag yielding the largest reduction (OR = 0.737, 26%), followed by the primary two-year specification (OR = 0.836, 16%), with the effect somewhat weaker at the shortest and longest windows examined. A cumulative spending measure, defined as the equal-weighted mean of spending across the preceding four years, also yielded a significant protective effect (OR = 0.760, 24% reduction in odds), meaning that sustained investment amplifies the protective association. The Symptomatic but Content profile showed a positive association with spending consistent with the main analysis across individual lag specifications and in the cumulative model (ORs ranging from 1.016 to 1.131). For the Vulnerable profile, no significant association was observed at any lag length or in the cumulative model, with odds ratios close to 1.0 and confidence intervals spanning both sides of the null throughout. These findings indicate that the differential pattern, that spending reduces risk of the most severe profile while leaving the hidden at-risk group unaffected, is not contingent on the two-year specification used in the primary analysis.

### Summary of key findings

The results can be summarized in five main findings. First, approximately half (51%) of the 9th-grade students demonstrated Complete Mental Health, but this proportion declined by 10% points from 2006 to 2024. Second, the Troubled profile (acute distress) more than doubled in prevalence over the study period, while the Vulnerable profile (low well-being without symptoms) remained stable at approximately 17%. Third, girls were substantially more likely than boys to be in all at-risk categories, and, more strikingly, this gender gap widened further over the study period, indicating a growing divergence between girls and boys rather than a stable difference. Fourth, increased municipal spending on school health services was associated with reduced odds of the most severe profile (Troubled; OR = 0.84, *p* = .007), but showed no comparable association with the Vulnerable profile (a borderline, non-significant estimate). Fifth, anxiety emerged as a differentiator between the Vulnerable and Troubled profiles, suggesting that students in the Vulnerable group may not be detected by symptom-based screening approaches.

## Discussion

This study examined whether municipal investment in school health services is associated with the distribution of dual-factor mental health profiles among adolescents in the Stockholm region. Among the findings summarized above, three are central to the interpretation that follows. First, we documented a substantial deterioration in adolescent mental health over the 18-year study period, with the proportion of students exhibiting Complete Mental Health declining by 10% points and the Troubled profile more than doubling in prevalence. Second, increased municipal spending on school health services was associated with reduced odds of the Troubled profile, characterized by both psychiatric symptoms and low well-being, but showed no significant association with the Vulnerable profile, characterized by low well-being without clinical symptoms. Third, spending was associated with increased odds of the Symptomatic but Content profile, suggesting that some students with severe symptomatology may experience improved well-being with greater service access, changing their profile classification rather than their symptom burden.

### Interpretation of main findings

By way of context, school health services in Sweden are statutorily required to work primarily preventively and health-promotively and to support students’ development toward the goals of education. In practice, provision combines routine health visits and screening at defined points with needs-triggered contacts for students who raise concern, and teams typically comprise nurses, physicians, psychologists, counsellors, and special educators. Importantly, resources are not currently allocated on the basis of dual-factor mental health profiles; the profiles used here are an analytical lens applied by us, not a planning tool used by municipalities. Our findings therefore describe how existing, largely symptom-oriented services relate to the distribution of profiles, and should be read as a critique of current allocation logic rather than an evaluation of a profile-based system.

The protective association of school health spending with the Troubled profile (OR = 0.84 per 1 000 SEK increase) is consistent with the symptom-focused orientation of most school-based mental health services. School nurses, psychologists, and counselors are trained to identify and respond to students presenting with psychiatric symptoms such as anxiety, sadness, and behavioral difficulties. When municipalities increase resources for these services, capacity for early identification and intervention expands, plausibly reducing the proportion of students progressing to the most severe mental health states. This interpretation aligns with evidence that school-based screening and psychological interventions represent cost-effective approaches to preventing mental disorders in young people [13].

However, the absence of a comparable association for the Vulnerable profile (OR = 1.08, 95% CI [0.99, 1.17], *p* = .084), an estimate of borderline significance, is equally important. These students, comprising 17% of the sample and remaining stable across the study period, report low well-being and poor school satisfaction but do not present with the psychiatric symptoms that typically trigger clinical attention. From a precision public health perspective [17], this finding suggests that current resource allocation strategies may systematically overlook a substantial subgroup of struggling students. The Vulnerable profile is not merely a milder form of distress; prior research demonstrates that these students show academic outcomes comparable to those with clinical symptomatology and may be at elevated risk for future mental health deterioration [10, 23]. Viewed from one angle, the association with the Troubled profile indicates that services are successfully reaching students with the greatest and most visible needs; the concern raised by our findings is therefore not with the students who are reached, but with a comparably sized group whose difficulties present as low school well-being without symptoms and who appear less likely to be reached by the same services.

The positive association with the Symptomatic but Content profile (OR = 1.09) is consistent with a partial compositional shift from the Troubled profile. Increased resources may help some students with severe symptomatology to sustain or recover a degree of well-being, moving them from the Troubled to the Symptomatic but Content category. This interpretation is consistent with the simultaneously observed protective effect on the Troubled profile and with evidence that school-based interventions can buffer emotional distress [5, 14].

### Temporal trends and gender disparities

The marked decline in adolescent mental health observed in this study mirrors international trends [1]. The acceleration of decline around 2014–2016, with the nadir occurring in 2022, suggests cohort-specific exposures that warrant further investigation. Potential contributors include increased social media use, academic pressure, international conflicts, climate anxiety, and, for the most recent waves, the COVID-19 pandemic and its aftermath.

The substantial gender gap, with girls nearly three times more likely than boys to be classified as Troubled by 2024, represents a critical public health challenge. That this gap widened over the study period suggests that whatever factors are driving the overall decline in adolescent mental health are affecting girls disproportionately. However, as adolescent boys are less likely to seek help for mental health problems, school health services may need to consider gender-specific approaches in both prevention and detection work [12, 39]. Several mechanisms may help explain why the deterioration has been steeper among girls. Girls report more intensive and more socially comparative use of social media, which has been linked to body dissatisfaction, sleep disruption, and depressive symptoms; they also tend to report greater worry about diffuse societal threats such as climate change and international conflict, and greater sensitivity to academic-performance pressure. Although our data cannot adjudicate between these explanations, the widening gap suggests that gendered exposures, rather than a stable difference between the sexes, are driving much of the recent decline and should be a priority for further research.

The stability of the Vulnerable profile across the study period, fluctuating narrowly between 15% and 19% even as other profiles shifted dramatically, is noteworthy. This consistency suggests that low well-being without psychiatric symptoms may represent a distinct developmental phenomenon rather than a prodromal state that inevitably progresses to clinical disorder. It also may indicate that the factors driving the overall mental health decline are primarily operating through the symptom pathway rather than the well-being pathway. Two further readings of this stability deserve consideration. First, some low school well-being in adolescence may reflect the normal ebb and flow of a developmentally turbulent period rather than incipient disorder; the survey items capture how students feel about school rather than clinical pathology, so the Vulnerable profile likely blends transient dissatisfaction with more persistent difficulty, reinforcing the need to avoid over-medicalizing the group. Second, and conversely, the stable prevalence of the Vulnerable group, even as symptomatic profiles rose sharply, argues against this pattern being merely noise: whatever the mix of causes, a consistent one-in-six share of students reports low school well-being that current services do not appear to shift.

### Implications for policy and practice

These findings carry several implications for the allocation of school health resources. First, the differential effects across profiles support a precision approach to school health investment [5]. Rather than asking whether spending “works” in aggregate, policymakers should consider which student subgroups benefit and which remain underserved. Our results suggest that current service models effectively reach students with visible psychiatric symptoms but are less likely to identify or support those whose struggles manifest primarily as diminished school well-being. A challenge here is to strike the right balance between supporting ‘Vulnerable’ students, without overmedicalizing normal ebbs and flows of adolescence.

Second, the persistence of the Vulnerable profile despite increasing investment points to a potential gap in service design. School health teams in Sweden and many other countries are mandated to work both preventively and health-promotively. Yet, the increase in psychiatric symptoms among students may in practice tilt school health services toward reactive, symptom-triggered intervention. Expanding the focus to include systematic assessment of well-being, and not merely the absence of symptoms, could help identify Vulnerable students who are likely to ‘fall through the cracks’.

Third, municipalities should anticipate that the apparent increase in the Symptomatic but Content profile may reflect a meaningful intermediate outcome: students whose distress has been partially ameliorated rather than a new or newly identified group. This reframes how to read prevalence shifts following investment increases; apparent growth in moderate-symptom categories may signal recovery from more severe states rather than rising need.

Fourth, the magnitude of effect, i.e., a 16% reduction in Troubled classification per €100 increase in per-student spending, provides a benchmark for cost-effectiveness considerations. If these patterns generalize nationally with roughly 6% classified as Troubled, even modest shifts in this proportion represent substantial numbers of adolescents. However, a full cost-effectiveness analysis would require data on downstream outcomes (healthcare utilization, educational attainment, labor market participation) that were beyond the scope of this study.

Finally, the finding that spending effects were concentrated in the central city of Stockholm warrants consideration. The greater Stockholm region comprises 26 municipalities and city-districts, with a diverse population and a comprehensive school health infrastructure that may enable more effective translation of resource investments into student outcomes. The stronger protective effect observed in central Stockholm city (OR = 0.72) compared to the full sample (OR = 0.84) suggests that the relationship between spending and mental health profiles may depend on organizational capacity to deploy resources effectively. In smaller municipalities, where school health teams may be more thinly spread and administrative structures less developed, additional funding may not yield the same benefits, or effects may be harder to detect given smaller student populations and less variation in spending over time. This interpretation aligns with research indicating that the effectiveness of health-promoting efforts depends not only on resource levels but also on how school leadership organizes and prioritizes this work [29]. From a policy perspective, these findings suggest that increasing school health budgets may be necessary but not sufficient; attention to implementation capacity and service organization may be equally important for translating investments into improved student mental health [28, 30]. We were not able to measure how individual municipalities organize their school health work (for example, staffing skill-mix, the balance between centralized and school-based provision, or the referral pathways in place), and these organizational differences were therefore not included in the models. It is consequently possible that some of the between-municipality variation we attribute to spending in fact reflects differences in how that work is organized and led, rather than the level of funding as such.

### Limitations and future research

The current study has strengths such as the large, population-based sample (*N* = 63 190) drawn from a consistent survey methodology over 18 years, the within-between decomposition of spending effects controlling for stable municipal characteristics that might confound the spending-outcome association, and the two-year lag between spending and outcomes reducing concerns about reverse causality. Yet, several limitations should be acknowledged. First, the observational design precludes causal inference. Although the within-municipality analysis controls for time-invariant confounders, time-varying confounders, such as changes in municipal socioeconomic composition or concurrent policy reforms, could bias estimates. Second, the spending measure captures aggregate municipal expenditure and cannot distinguish between different service components (e.g., nursing vs. psychology) or service quality, nor can it isolate the share devoted specifically to students’ mental health, since school health services also address physical health and learning support. Two municipalities with identical per-student spending may deliver very different services. Third, the well-being items in this study were school-specific (e.g., “I thrive at school”) rather than assessing global life satisfaction. While school well-being is highly relevant for school-based services, the Vulnerable profile as identified here may not fully correspond to the construct as defined in broader dual-factor research. Fourth, Stockholm municipality comprised 90% of the sample, and robustness analyses revealed that the protective effect on the Troubled profile was primarily driven by this municipality. The Stockholm-only analysis showed a stronger effect (OR = 0.72, *p* < .001) than the full sample, while the effect was not significant when Stockholm was excluded (OR = 0.97, *p* = .813). This pattern may reflect limited statistical power in the non-Stockholm subsample (*n* = 6 265), genuine heterogeneity in spending effects between large urban and smaller suburban municipalities, or differences in how school health services are organized and delivered across municipal contexts. The findings may therefore be most applicable to large urban municipalities with similar service structures to Stockholm. Future research should examine whether spending effects generalize to other Swedish regions with different demographic and organizational characteristics. Fifth, we could not examine precise theoretical mechanisms, i.e., the pathways through which spending might influence profiles, nor potential moderators such as gender or socioeconomic background. Relatedly, we did not adjust for socioeconomic status at the municipal level. Beyond the over-control concern noted above (socioeconomic disadvantage is an explicit input to the needs-based funding formulas that generate the exposure), municipal averages would in any case be a poor proxy for the construct of interest: the socioeconomic variation that matters operates at the school and neighbourhood level, and Stockholm municipality, which contributes about 90% of the sample, is itself highly heterogeneous, spanning districts across the full socioeconomic range. Because the data identify each student’s municipality but not their school, school-level socioeconomic status (which is published by the Swedish National Agency for Education) could not be linked, and aggregating it to the municipality would average away precisely the variation of interest. A further limitation concerns representativeness: because the survey is administered in class, students who are frequently absent may be underrepresented, and if absence is itself associated with distress, the prevalence of the most severe profiles may be understated.

Several directions for future research emerge from these findings. First, studies examining underlying mechanisms are needed: does school spending operate through increased screening, more counseling hours, faster referral to specialist services, or improved school climate? Second, research should examine whether the benefits of spending on school health services differ across student subgroups defined by e.g., gender, socioeconomic background, or ethnic background. Third, intervention studies specifically targeting the Vulnerable profile – perhaps through universal well-being promotion rather than selective symptom-based programs – could test whether this group is amenable to school-based support. Fourth, longitudinal designs tracking individual students across time could examine whether spending on school health services influences transitions between student profiles (e.g., preventing movement from Vulnerable to Troubled). Finally, cost-effectiveness analyses incorporating longer-term outcomes would inform resource allocation decisions, and comparative studies that measure how municipalities organise their school health work could help disentangle the effect of funding level from that of service organisation and leadership.

## Conclusion

This study provides new evidence that municipal spending on school health services is associated, and associated unequally, with distinct mental health profiles among adolescents. Increased spending was associated with a lower prevalence of the most severe profile (students with both psychiatric symptoms and low school well-being) but showed no comparable association for students whose difficulties manifest as diminished school well-being without clinical symptoms. From a public health perspective, these findings suggest that current service models, while valuable, may require reorientation to address the full spectrum of adolescent mental health needs. As policymakers respond to the ongoing decline in youth mental health, attending to heterogeneity in treatment effects, asking not just “does it work?” but “for whom?“, will be essential for efficient and equitable resource allocation.

## Data Availability

The Stockholm survey data analyzed during the current study are not publicly available due to data protection regulations governing anonymized survey data collected from minors but are available from the City of Stockholm on reasonable request, subject to a data-sharing agreement. Municipal spending data on school health services are publicly available from the Swedish National Agency for Education (Skolverket) at https://www.skolverket.se. The Stata and Mplus code used to construct analytical variables and produce the reported analyses is available from the corresponding author on reasonable request.

## Abbreviations

Abbreviation: Full term
CI: Confidence interval
FIML: Full information maximum likelihood
gsem: Generalized structural equation modeling
LPA: Latent profile analysis
M: Mean
MLR: Maximum likelihood with robust standard errors
MH: Mental health
N: Total sample size
n: Subsample size
OR: Odds ratio
r: Pearson correlation coefficient
WHO: World health organization
